# Tracking Native *Tetrahymena* Ribozyme Folding with Fluorescence

**DOI:** 10.1021/acs.biochem.3c00363

**Published:** 2023-11-01

**Authors:** Jeffrey P. Potratz, Rick Russell

**Affiliations:** † Department of Molecular Biosciences, University of Texas at Austin, Austin, Texas 78712, United States; ‡ Department of Physical Sciences, Concordia University Wisconsin, 12800 North Lake Shore Drive, Mequon, Wisconsin 53097, United States

## Abstract

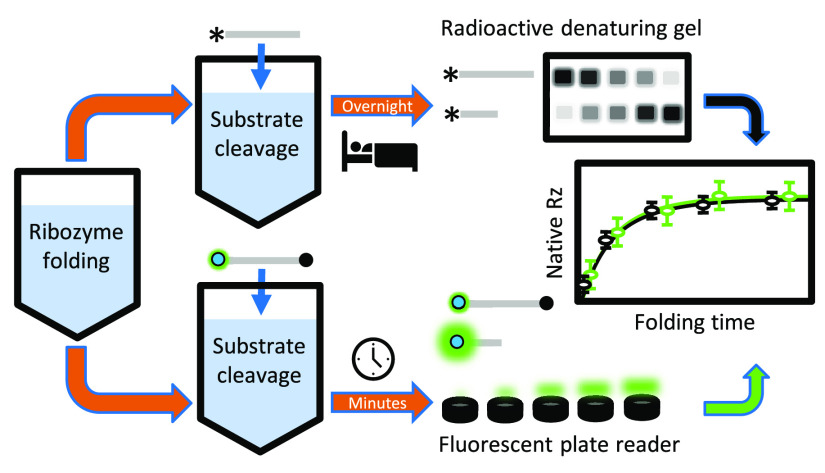

Folding of the *Tetrahymena* group I intron ribozyme and other structured RNAs has been measured using a catalytic activity assay to monitor the native state formation by cleavage of a radiolabeled oligonucleotide substrate. While highly effective, the assay has inherent limitations present in any radioactivity- and gel-based assay. Administrative and safety considerations arise from the radioisotope, and data collection is laborious due to the use of polyacrylamide gels. Here we describe a fluorescence-based, solution assay that allows for more efficient data acquisition. The substrate is labeled with 6-carboxyfluorescein (6FAM) fluorophore and black hole quencher (BHQ1) at the 5′ and 3′ ends, respectively. Substrate cleavage results in release of the quencher, increasing the fluorescence signal by an average of 30-fold. A side-by-side comparison with the radioactivity-based assay shows good agreement in monitoring *Tetrahymena* ribozyme folding from a misfolded conformation to the native state, albeit with increased uncertainty. The lower precision of the fluorescence assay is compensated for by the relative ease and efficiency of the workflow. In addition, this assay will allow institutions that do not use radioactive materials to monitor native folding of the *Tetrahymena* ribozyme, and the same strategy should be amenable to native folding of other ribozymes.

The onset of catalytic activity has long been leveraged as a signal for productive folding to the native conformation by a class of large, catalytically active RNAs known as ribozymes. Unlike other experimental approaches, the use of catalytic activity can clearly and decisively differentiate between a functional, native conformation and a conformation that contains numerous native structural elements but is non-native. Only the native conformation can cleave an oligonucleotide substrate. This approach has been used with the hammerhead ribozyme, group I and II introns, ribozymes derived from group I and II introns, and with RNA chaperone proteins present as well.^1−4^

The folding process of the *Tetrahymena* ribozyme, derived from a group I intron, has been studied for decades to gain insight into how a large, structured RNA folds into its native state. Folding proceeds through a series of structured intermediates, including a long-lived misfolded conformation that is highly compact, includes extensive native structure, but differs from the native structure in its topology and is required to unfold extensively to ultimately reach the native conformation.^5−9^ This misfolded conformation is populated by 90% of the ribozyme during folding *in vitro*, and its refolding transition to the native conformation has been valuable for understanding complex RNA folding processes and how RNA chaperone proteins assist in RNA folding.^10−12^ Recent cryo-EM structures of the native and misfolded ribozyme^13−15^ confirmed previous conclusions from biochemical work that the misfolded ribozyme has extensive native structure but differs in topology.^7,8^ The structures extend the understanding by establishing the specific topological change and hypothesizing folding pathways for formation and resolution of the misfolded conformation.

Much of the biochemical work on the folding transitions of this ribozyme used the onset of catalytic activity as an experimental readout for formation of the native state.^3^ The standard assay used to observe and quantify this catalytic activity relies on monitoring the cleavage of a radiolabeled oligonucleotide.^5^ Most commonly, the 11-nucleotide substrate is labeled with ^32^P at the 5′ end, and the 6-nt cleavage product is separated from the substrate using denaturing polyacrylamide gel electrophoresis. Quantitation of the band intensities reveals the fraction of the substrate that has been cleaved to the product, which reports on the fraction of ribozyme in the native state.

This radioactivity-based assay has been used successfully for decades and was used to discover the prominent misfolded conformation.^5^ The staple assay using ^32^P and denaturing gels is well established and has intrinsic strengths. Because subnanomolar substrate concentrations are used, it permits the use of nanomolar ribozyme concentrations, which allows correspondingly low concentrations of chaperone proteins to be added to probe for concentration-dependent effects.^12^ The assay also produces an experimental signal, the fraction of substrate cleaved into product, that serves as a direct readout for the fraction of native ribozyme and mitigates effects of loading slightly different volumes of quenched cleavage reactions on the gel. However, there are also inherent limitations of the assay. Its use of radioactivity necessitates extra safety precautions, meticulous administrative documentation, and additional labor as demonstrated by reoccurring labeling of the substrate due to the short half-life of ^32^P. Additionally, its use of a gel and phosphor screen for observing substrate cleavage requires an overnight waiting step when working with amounts of radioactivity meant to limit the exposure of the researcher, as is common practice.

Here we describe the development and validation of an assay that is solution-based instead of gel-based and uses fluorescence rather than radioactivity. The assay uses an increase in fluorescence resulting from substrate cleavage and the corresponding loss of a quencher, and we apply the assay to track native folding of the *Tetrahymena* ribozyme. The solution-based assay allows for more efficient data acquisition, and because the assay uses fluorescence instead of radioactivity, it is more broadly accessible to researchers. The folding rate constants obtained with the fluorescence assay are comparable to those obtained with the standard radioactivity-based assay, but side-by-side measurements show that there is reduced precision relative to the radioactivity-based assay. The general strategy of the assay should be suitable for use with other ribozymes as well.

## Materials and Methods

### Preparation and Storage of RNA

The L-21/ScaI form of the *Tetrahymena* ribozyme was transcribed using a HiScribe T7 High Yield RNA Synthesis Kit (New England Biolabs, NEB) and purified using RNeasy columns (Qiagen). Ribozyme concentration was determined by spectrophotometry using an extinction coefficient of 3.9 × 10^6^ M^–1^ cm^–1^ at 260 nm. Substrate oligonucleotides were purchased from Integrated DNA Technologies (IDT). rSA_5_ (CCCUCUAAAAA) was ordered with standard desalting purification, 5′-labeled with [γ-^32^P] (PerkinElmer) using T4 polynucleotide kinase (NEB), and purified on a 20% denaturing polyacrylamide gel. 6FAM-rSA_5_-BHQ1 (6-FAM/CCCUCUAAAAA/BHQ1) and 6FAM-rP (6-FAM/CCCUCU) were ordered with HPLC purification, resuspended in TE buffer, and quantified with absorbances at 260 nm using extinction coefficients of 138 160 M^–1^ cm^–1^ and 67 460 M^–1^ cm^–1^, respectively. Solutions of 6FAM-rSA_5_-BHQ1 and 6FAM-rP were wrapped in aluminum foil to protect the fluorophore from photobleaching.

### Reaction Conditions

The ribozyme (56–3333 nM) was misfolded by incubation in 50 mM Na-MOPS pH 7.0 with 10 mM MgCl_2_ or 10 mM CaCl_2_ at 25 °C for 5 min. Alternatively, it was folded to the native state by incubation at 50 °C for 30 min in 50 mM Na-MOPS (pH 7.0) with 10 mM MgCl_2_. Misfolded ribozyme was diluted to concentrations of 17–1000 nM in 3, 5, or 7 mM Mg^2+^ at 25 °C with 50 mM Na-MOPS pH 7.0, 2 mM ATP-Mg^2+^, and CYT-19 buffer (5% glycerol, 50 mM KCl, 2 mM Tris pH 7.5, 100 μM EDTA, 20 μM DTT), included for consistency with other published work,^12,16,17^ and time points were added to the cleavage reaction. The cleavage reaction was performed at 25 °C or room temperature (RT) and contained 10–600 nM ribozyme, 49.8–52.2 mM MgCl_2_, 500 μM guanosine, 0.5 mg/mL Proteinase K, and other solutes present in the folding reaction at 60% of their initial concentrations. 6FAM-rSA_5_-BHQ1 or radiolabeled substrate was added to initiate the cleavage reaction and was present in final concentrations of 5–150 and ≤1 nM, respectively. Ribozyme controls folded with Ca^2+^ or to the native state bypassed the folding stage and were added directly to a cleavage reaction containing Ca^2+^ or Mg^2+^, respectively. These cleavage reactions contained the same solution conditions as above. Cleavage time points were quenched with pH 8.0 EDTA solutions to final concentrations of 67–85 mM. Single cleavage time points were taken at 1 min for reactions with 600 nM ribozyme, 1.5 min for reactions with 30–60 nM ribozyme, and 9 or 10 min for reactions with ≤12 nM ribozyme.

### Radioactivity and Fluorescence Measurements

Radiolabeled substrate and product were separated on a 20% denaturing polyacrylamide gel and quantified using a phosphorimager and ImageQuant TL instrument (GE Healthcare). Quenched cleavage reactions with fluorescent oligonucleotides were either loaded in a low volume 384-well black flat bottom polystyrene NBS microplate (Corning) and quantified using a Tecan Spark 10M plate reader or loaded in a low volume 384-well black round-bottom polystyrene NBS microplate (Corning) and quantified using a SpectraMax M5 plate reader (Molecular Devices). Both plate readers utilized monochromators and the top module reads. Excitation and emission wavelengths of 485 and 530 nm, respectively, were used. Settings on the Spark 10M were the default 30 flashes, the default 40 μs integration time, the 510 dichroic mirror, the z-position calculated from a well containing sample, and an optimal gain. Settings on the SpectraMax M5 were the default six flashes with automatic gain. Microplates were loaded with 10 or 20 μL. The average relative fluorescence unit (RFU) value of three or more blanks containing the reaction matrix, absent any fluorescent oligonucleotides, was subtracted from all sample RFU values.

### Data Fitting

RFU values from cleavage reactions were converted into the fraction product. RFU values were normalized by an average value from triplicate FC (folded control) reactions, signifying 100% native ribozyme. The data were then multiplied by the active fraction of ribozyme present (∼0.9) as determined by triplicate FC reactions in the radioactivity-based assay. Rate constants for cleavage time courses were determined by fitting the time-dependent increase of the fraction product with a single exponential equation (Kaleidagraph, Synergy Software). Rate constants are reported as the average and standard error of at least three independent determinations. The plots of increasing simulated and observed rates of cleavage as a function of increasing ribozyme concentration were fit with a hyperbola.

RFU values from folding reactions with fluorescent substrate and fraction product values from folding reactions with radiolabeled substrate were converted into fraction native ribozyme by subtracting a value from SC+ (substrate control plus Ca^2+^-folded ribozyme) reactions, signifying 0% native ribozyme, when available. The data were then normalized by a value from FC (folded control) reactions signifying 100% native ribozyme, when available. In the absence of controls, the data were normalized by the maximum observed RFU value. Rate constants for folding time courses were determined by fitting the time-dependent increase of fraction native with a single exponential equation. The y-intercept was allowed to float, and the end point was fixed to 1. Rate constants are reported as the average and standard error of at least three independent determinations.

## Results

### Discontinuous Assay Overview

Folding of the *Tetrahymena* ribozyme from the long-lived misfolded state to the native state has long been assessed by a discontinuous assay, in which the folding occurs in the first stage and substrate cleavage (catalysis) occurs in the second stage.^3^ Aliquots from the folding reaction (stage 1) are removed at various times to measure substrate cleavage (stage 2). Further refolding of the misfolded ribozyme during catalysis is blocked by including a high Mg^2+^ concentration (50 mM final). Aliquots removed from the folding stage contain increasing fractions of native ribozyme with increasing folding time, reflecting the refolding of the misfolded ribozyme. The substrate cleavage reaction is initiated for each aliquot by the addition of limiting, labeled substrate and the cofactor guanosine (500 μM final). Substrate cleavage is stopped by addition of EDTA (∼80 mM final) in excess of Mg^2+^, as Mg^2+^ is required for catalysis of cleavage. The refolding reaction is often carried out with low Mg^2+^ concentrations (≤10 mM) and in the absence of any protein to study the rearrangement.^6,7^ The addition of RNA chaperone proteins to the folding stage has also helped elucidate strategies employed by chaperone proteins.^10−12,18^ Proteinase K present in the substrate cleavage reaction degrades any chaperone proteins present in the folding reaction.^6,7^

**Figure 1:**
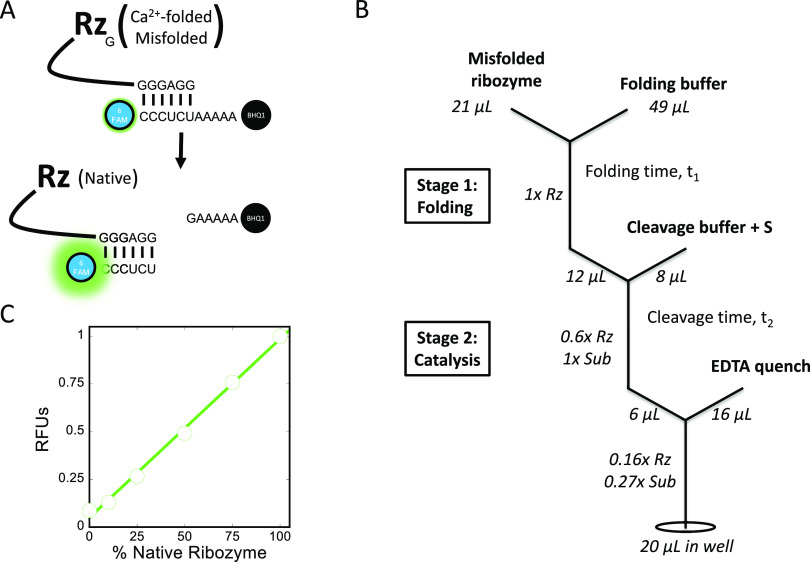
Assay overview. (A) The fluorescent substrate can only be cleaved by ribozyme folded to the native state, resulting in an increased fluorescence. (B) The reaction schematic highlights the stages of the discontinuous assay. (C) The relative level of fluorescence signal from the substrate increases linearly in control reactions with increasing percentages of native ribozyme: 0% *N* = SC+ (substrate control plus Ca^2+^-folded ribozyme), 10% *N* = MC (misfolded control), 100% *N* = FC (folded control), 25, 50, and 75% *N* = mixtures of MC and FC. Displayed RFU values are averages and standard errors of three cleavage reactions that were normalized by the average FC RFU value. Concentrations of 60 nM ribozyme and 50 nM substrate were used.

In this assay, the results of the substrate cleavage reactions indicate the fraction of native ribozyme in the following way. The cleavage reactions are single turnover with ribozyme in excess of substrate, so the limiting substrate can bind to either the native or misfolded ribozyme. Both ribozyme forms bind substrate with nearly the same rate constant, as they can both form base pairs with the substrate, but only the native ribozyme cleaves the substrate^6^ (Figure 1A). Thus, the single turnover reaction displays a fast exponential rise in the fraction of product with an amplitude approximately equal to that of the fraction of native ribozyme. A slower phase of substrate cleavage, on the time scale of hours and not measured here, reflects substrate dissociation from the misfolded ribozyme and subsequent binding to the native ribozyme. Once the cleavage reaction time courses have established the time necessary to reach the end of the fast exponential rise, a single time point from the cleavage reaction is sufficient to report on the fraction of ribozyme in the native state. A schematic of the discontinuous assay detailing the folding and cleavage stages is shown in Figure 1B.

### Fluorescence Assay Development

The traditional radioactivity-based assay uses a substrate that is ^32^P-labeled at the 5′ end. For the fluorescence assay, we used a substrate of the same nucleotide sequence (CCCUCUA_5_) that was labeled at the 5′ end with 6FAM and at the 3′end with BHQ1. Ribozyme-mediated cleavage of this substrate is expected to result in rapid release of the quencher, along with the cofactor and nucleotides downstream of the cleavage site (GA_5_) and, therefore, an increase in fluorescence signal.

To convert a fluorescence signal to a corresponding fraction of native ribozyme, it was necessary to establish benchmark fluorescence levels corresponding to zero native ribozyme and 100% native ribozyme. To determine the fluorescence level in the absence of any substrate cleavage (i.e., corresponding to the absence of native ribozyme), we performed a control reaction where the substrate is bound to ribozyme folded in Ca^2+^ (SC+, substrate control plus Ca^2+^-folded ribozyme), which does not support the cleavage reaction (Figure 1A, top).^19^ Analogously, to determine the maximum signal, we performed a control reaction in which the ribozyme is prefolded to the native state (FC, folded control; Figure 1A, bottom). Other control reactions with varying native ribozyme percentages were performed by combining aliquots in which the ribozyme was prefolded to ∼90% of the long-lived, misfolded conformation (MC, misfolded control) with FC aliquots. These control reactions revealed a linear increase in fluorescence with an increase in native ribozyme (Figure 1C). Together, these results show that there is a robust fluorescence signal upon substrate cleavage, and the extent of this change provides a good measure of the fraction of native ribozyme.

### Cleavage Reaction

**Figure 2:**
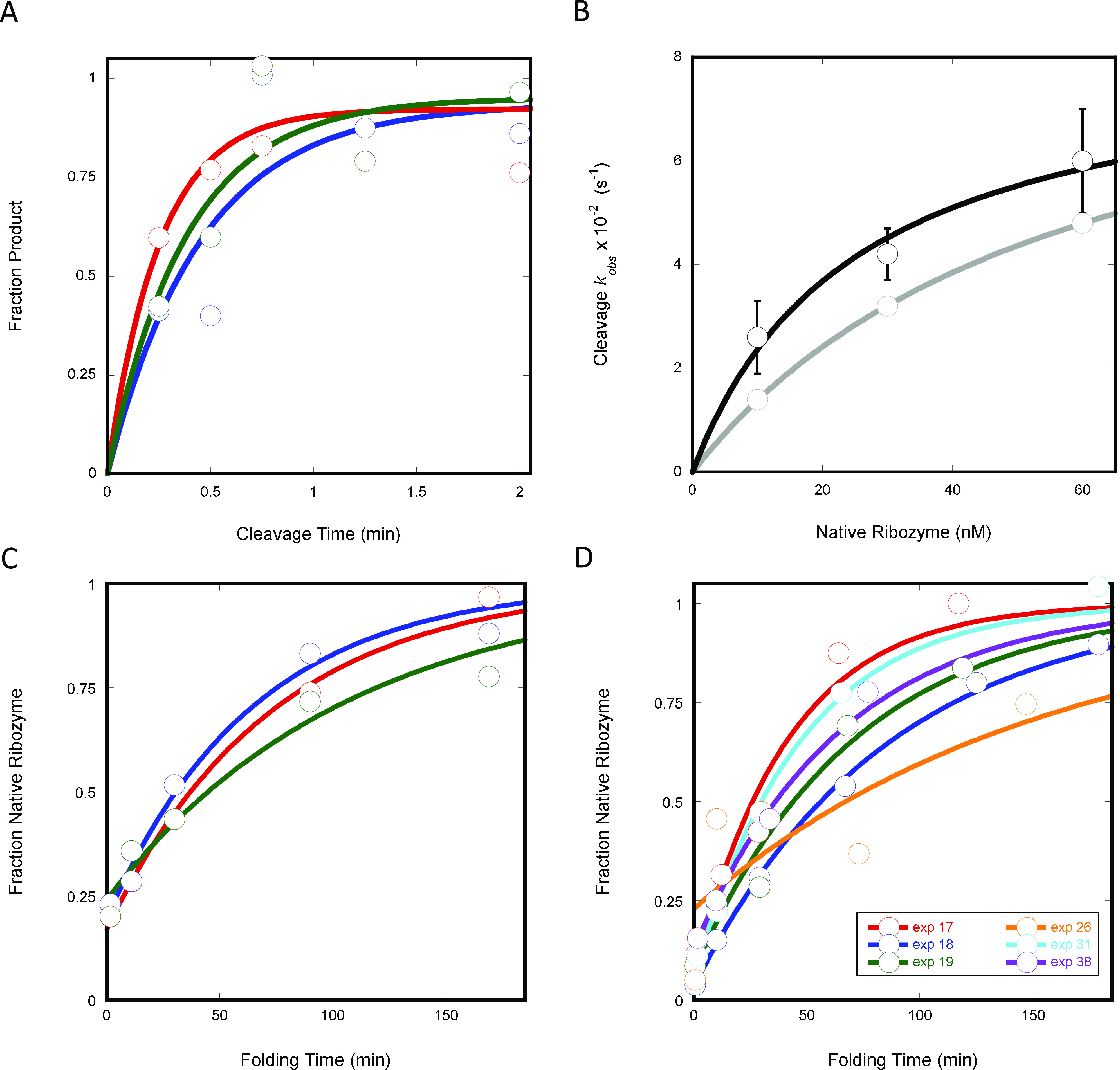
Substrate cleavage and ribozyme folding reaction time courses. (A) Triplicate cleavage reactions (red, green, blue) with 60 nM ribozyme and 50 nM substrate. (B) Comparison of predicted cleavage rates from simulation (gray) and those observed (black). Published rate constants for substrate binding to ribozyme,^20^ 1.7 × 10^6^ M^–1^ s^–1^, and cleavage by ribozyme,^7^ 0.17 s^–1^, were input into Kinetic Explorer v.10 simulation software (Kintek) along with initial ribozyme and substrate concentrations present in experiments. Simulated product time traces were fit with an exponential to obtain the predicted *k*_*obs*_ values. (C) Folding reactions with 10 nM ribozyme and 5 nM substrate in 3 mM Mg^2+^ showing replicate reactions from the same day and (D) with 60 nM ribozyme and 50 nM substrate in 3 mM Mg^2+^ on different days.

To determine whether the fluorescent substrate behaved similarly to the radiolabeled substrate, without interference from the fluorophore at the 5′ end and quencher at the 3′ end, cleavage time courses were monitored (Figure 2A). We found that the observed rates of cleavage with the fluorescent substrate agreed with predicted values based on simulations using both known binding and cleavage rate constants obtained with the radiolabeled substrate (Figure 2B).

As mentioned above, once the time required to reach the end of the exponential increase of product formation in the cleavage stage is known, it is necessary to take only one time point per cleavage reaction to assess the fraction of the native ribozyme. This knowledge allowed the use of a single cleavage time point per cleavage reaction, taken at 1.5 min in reactions with 60 nM ribozyme. Importantly, the concentration of ribozyme in the cleavage reaction (which is in excess over substrate) dictates when the exponential increase of product is over, and the single time point should be taken.

### Folding Reaction

**Figure 3:**
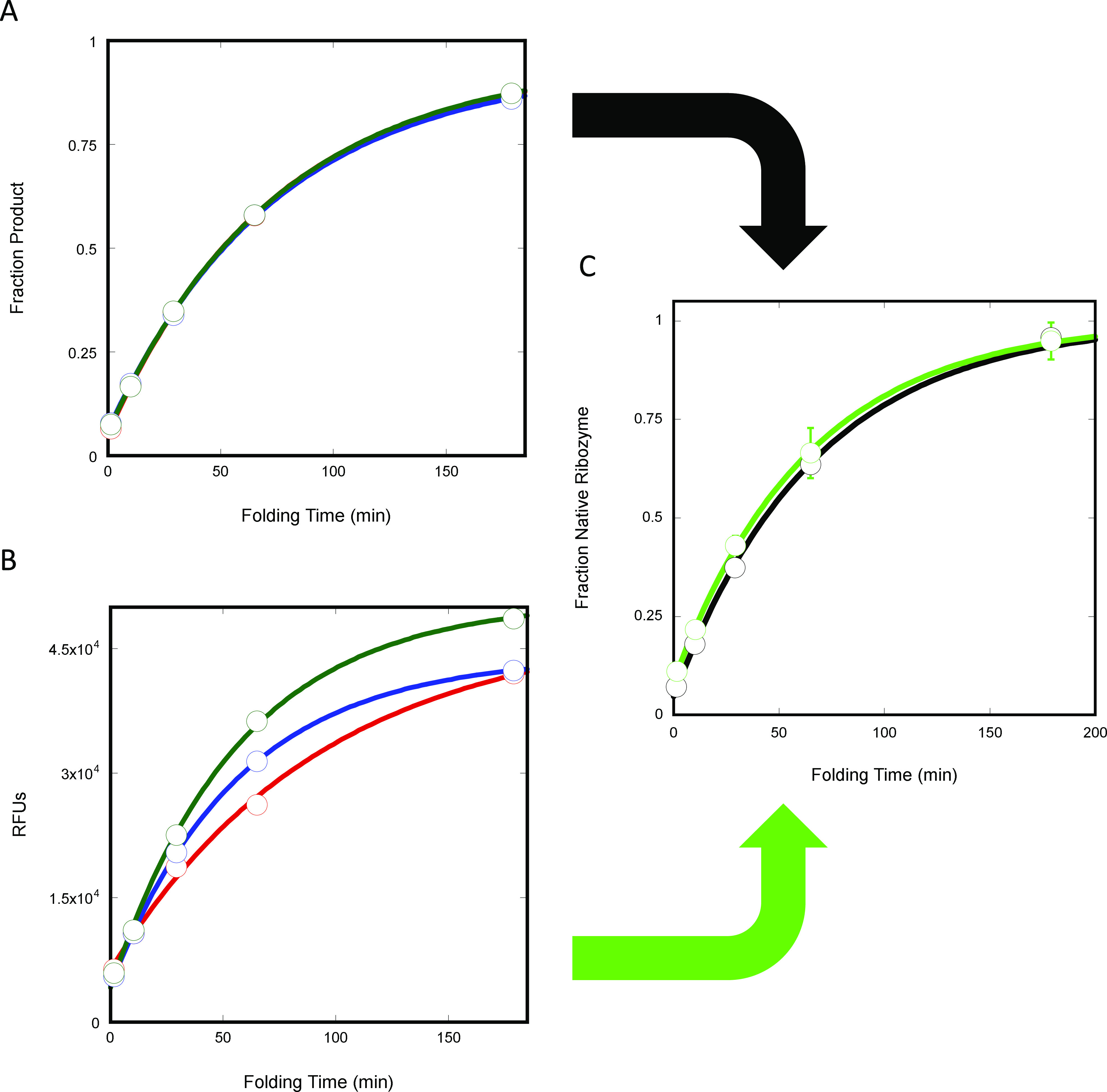
Fluorescence assay validation. Triplicate ribozyme folding time courses at 3 mM Mg^2+^ with (A) 60 nM ribozyme and ≤1 nM radiolabeled substrate (blue, red, green) and (B) 60 nM ribozyme and 50 nM fluorescent substrate (blue, red, green). Data from the radioactivity-based assay (A) and the fluorescence assay (B) were converted into fraction native ribozyme (C). An average value from triplicate SC+ (substrate control plus Ca^2+^-folded ribozyme) reactions signifying 0% native ribozyme was subtracted, and the corresponding values were normalized by an average value from triplicate FC (folded control) reactions signifying 100% native ribozyme. In C, triplicate radioactivity-based assay data (black) time points were displayed as the average and standard error, and the fluorescence data (bright green) were displayed similarly for comparison.

Folding reactions starting from the long-lived misfolded state were used to test whether the assay could provide reproducible folding rate constants. Reproducibility of the assay was evaluated from triplicate folding curves obtained in the same experiment (Figures 2C and 3B) and from folding curves obtained in experiments run on different days (Figure 2D and Table 1).

**Table 1:** Reproducibility of Folding with Fluorescence Assay at 3 mM Mg^2+^

	Same Day Rxns	Same Day Rxns	Different Day Rxns	All Rxns
*k*_*obs*_ × 10^–4^, s^–1^	2.7 ± 0.5	2.1 ± 0.3	2.6 ± 0.4	2.8 ± 0.3
[Substrate], nM	50	5	50	5–150 nM
*N*	3	3	6	23

### Fluorescence Assay Validation

The assay was validated by comparing results obtained with it to results obtained with the standard radioactivity-based assay (Figure 3C and Table 2). Rate constants obtained with each assay were averaged, and the differences between rate constants were divided by the average and converted to a percent. Reactions were carried out as detailed in the schematic above (Figure 1B) except with increased volumes. This allowed sufficient folding volumes, so each 100 nM ribozyme folding time point could be split into two distinct cleavage reactions, one which included ≤1 nM radiolabeled substrate and one which included 50 nM fluorescent substrate.

**Table 2:** Fluorescence Assay Validation by Comparison to the Standard ^32^P Assay

[Mg^2+^], mM	Fluorescence Assay *k*_*obs*_, s^–1^	^32^P Assay *k*_*obs*_, s^–1^	% Difference
3	(2.7 ± 0.5) × 10^–2^	(2.50 ± 0.03) × 10^–2^	8
5	(5.3 ± 0.5) × 10^–5^	(4.25 ± 0.04) × 10^–5^	22
7	(1.48 ± 0.06) × 10^–5^	(1.13 ± 0.04) × 10^–5^	27

### Measurement of Substrate Detection Limit

With the fluorescence assay giving reproducible folding results (Table 1) that agreed well with the standard radioactivity-based assay (Table 2), we explored the minimum substrate concentration that would give a reliable experimental signal. The detection limit was probed using the 6FAM-rP oligonucleotide, which mimics the 5′ product that remains bound to the ribozyme (Figure 1A bottom) and Ca^2+^-folded ribozyme. A mock “cleavage” reaction with 0.1 nM 6FAM-rP gave RFU readings just above blank values. After adjusting for dilution with EDTA quench solution and total volume of solution, this corresponds to a concentration of 27 pM and an amount of 0.5 fmoles in the microplate well. This is in reasonable agreement with the Tecan Spark 10M plate reader manual that states a detection limit of <20 pM for fluorescein, the fluorophore from which 6FAM is derived. Folding time courses performed with as low as 5 nM 6FAM-rSA_5_-BHQ1 substrate gave reproducible folding rate constants that were in good agreement with reactions using 50 nM substrate or greater (see Figure 2C and Table 1).

## Discussion

### Fluorescence Assay Bypasses the Limitations of the Radioactivity-Based Assay

In developing a fluorescence assay to follow *Tetrahymena* ribozyme folding by catalytic activity, we sought to design it such that it would avoid the limitations of the radioactivity-based assay and would come as close as possible to duplicating the strengths of the radioactivity-based assay. By definition, the fluorescence-based assay avoids key limitations of the radioactivity-based assay, as it removes the requirements for administrative burdens and radioactivity-specific safety precautions. In addition, the assay avoids gels and phosphor screens, decreasing the number of hands-on steps and drastically reducing the amount of overall time needed to obtain data from a folding experiment from next-day results with the radioactivity-based assay to same-day results with the fluorescence assay.

### Comparison to the Strengths of the Radioactivity-Based Assay

The principal strengths of the staple radioactivity-based assay are the high sensitivity, allowing the use of subnanomolar substrate concentrations and therefore low nanomolar ribozyme concentrations, and the direct readout of the fraction of the substrate cleaved. It should be noted that low nanomolar ribozyme concentrations are not usually necessary for folding experiments but are helpful under some circumstances, such as when using a tight-binding chaperone protein. We found that the fluorescence-based assay is workable with substrate concentrations as low as 5 nM. Importantly, this value allows use of ribozyme and chaperone protein concentrations in the folding stage that are as low as any used with the radioactivity-based assay.^12^ Additionally, the precision of folding rate constants is similar using either 50 or 5 nM substrate (Table 1) so long as ribozyme is in excess. Attaining this level of precision with 5 nM substrate agrees well with the detection limit of the assay being near 0.1 nM product. These values correspond to being able to measure the folding reaction with 2% resolution. That is the assay can theoretically detect when as little as 2% of the substrate has been cleaved.

In the fluorescence assay, substrate cleavage releases a quencher and produces an increased fluorescence. To obtain the fraction of substrate cleaved, this fluorescence signal (in RFUs) must be converted to a fraction through comparison with the RFU value of an FC control reaction. In contrast, the radioactivity-based assay produces signals for both the substrate and product from each time point such that differences in the volume loaded do not contribute to uncertainty in the fraction of substrate cleaved. This difference between the assays undoubtedly contributes to the lower level of precision observed with the fluorescence assay (Table 2). Interestingly, the 7 mM Mg^2+^ folding reactions are the most precise of the fluorescence assays but also have the largest percent difference when compared to the radioactivity-based assay. These features are most likely the result of the folding process being only ∼25% complete with 7 mM Mg^2+^ at the last folding time point and requiring extrapolation to complete folding (Figure S1F). Specifically, the end points of the exponential fits were dictated by values obtained in control reactions, where the ribozyme was fully folded to the native state (FC). Monitoring complete folding at 7 mM Mg^2+^ would require time points being taken over the course of days. If this strategy was employed, then best practice would be waiting to perform all cleavage reactions at the same time after all folding time points have been collected. This method ensures all fluorescent substrates and products are exposed to essentially the same amount of light and that pipet volumes are set exactly the same for each cleavage reaction. This general approach has produced more internally consistent folding curves when compared to experiments in which cleavage reactions were run while waiting to collect the final folding time point followed by the final cleavage reaction being performed after collection.

### Potential Applications of the Fluorescence Assay

The fluorescence assay is ready-for-use to track native folding of *Tetrahymena* ribozyme. Experimental conditions are laid out (see protocol below, Figures 1B and S2), and control reactions (SC+ and FC) used to set the floor and ceiling of folding reactions have been established. These control reactions allow vastly different magnitudes of RFU values (see Figure 3B data with Tecan Spark 10M and Figure S3 data with Molecular Devices SpectraMax M5) to be normalized to fractional native ribozyme values that can be directly compared. When monitoring a complete folding curve, the assay gives results that are only 8% different from the standard radioactivity-based assay and in roughly half the time.

It might be possible to improve the fluorescence assay by using an internal standard to control for any variability in the loading volumes in the microplate. This internal standard could be a fluorophore with excitation and emission wavelengths distinct from (probably longer than) the excitation and emission wavelengths of the fluorescent substrate. A solution containing the internal standard and a 10× substrate concentration would be added to initiate cleavage reactions. Product RFU values of each well would be normalized by the RFU values of the internal standard from the corresponding wells. If successful, this approach would minimize variability, resulting from small differences in loading volumes. However, it is not clear whether the variability in loading volumes is greater than the variability that would result from two separate plate reads utilizing two distinct excitation and emission wavelength pairs. This approach was not necessary here, as the large fluorescence change upon substrate cleavage (30-fold) was sufficient to achieve reliable results that were comparable to those of the radioactivity-based assay, but it may prove useful for other applications with smaller signals or increased variability.

The general approach to the fluorescence assay should be amenable for use with other ribozymes. Care should be taken when selecting the location of the fluorophore and quencher on the substrate. The substrate used in this study was short enough to allow robust quenching with attachment of both to opposite ends of the oligonucleotide, improving the likelihood that they would not interfere with binding or catalysis. The fluorophore and quencher were chosen due to their cost-effectiveness and widely applicable excitation and emission wavelengths for use with plate readers. The assay relies on catalytic activity of the ribozyme to separate the substrate into two products, one with a fluorophore and one with quencher. Dissociation of at least one of these products results in a large increase in fluorescence signal. None of these characteristics are exclusive to the *Tetrahymena* ribozyme. Thus, the assay should be applicable to other ribozymes, as well.

## Step-by-Step Protocol

For the overall assay scheme, please refer to Figure 1B. For a more detailed schematic, please refer to Figure S2.

(1) Prepare a solution of 3.33× concentration of *Tetrahymena* ribozyme in 10 mM MgCl_2_ and 50 mM Na-MOPS pH 7.0. Incubate a portion of this solution at 50 °C for 30 min to fold the ribozyme to the native state. (3.6 μL of native ribozyme is needed for each FC cleavage reaction, so a 12 μL aliquot provides sufficient volume for triplicate FC reactions.) Incubate the remainder of the solution at 25 °C for 5 min to fold the ribozyme to the misfolded state. (21 μL of misfolded ribozyme is needed to provide each folding reaction with sufficient volume for five time points.) Store both ribozyme solutions on ice.
(2) Mix 21 μL of misfolded ribozyme with 49 μL of folding buffer solution (i.e., 1 volume to 2.33 volumes) to initiate folding and obtain a 1× concentration of ribozyme. The folding buffer contains enough Na-MOPS pH 7.0 and MgCl_2_ to obtain final concentrations of 50 mM Na-MOPS and the desired Mg^2+^ concentration, bearing in mind the contribution of these components from the misfolded ribozyme portion. (The folding buffer only contains 4.9 μL of 500 mM Na-MOPS pH 7 so that, after mixing with misfolded ribozyme, the final concentration is 50 mM.) The folding buffer also contains ATP-Mg^2+^ and chaperone protein (or protein buffer) to arrive at 2 mM ATP-Mg^2+^ and the desired protein concentration after mixing with misfolded ribozyme. If a chaperone protein is used, add it to the folding buffer 30 seconds before mixing the folding buffer with the misfolded ribozyme. Mixing of the misfolded ribozyme and the folding buffer initiates the start of folding (folding time, *t*_1_ = 0).
(3) Add folding time points (12 μL) to cleavage buffer (6 μL) containing sufficient concentrations of MgCl_2_, guanosine, and Proteinase K such that after 6FAM-rSA_5_-BHQ1 substrate is added (2 μL) the final concentrations are 50 mM, 500 μM, and 0.5 mg/mL, respectively. However, initially the substrate is absent, and the high Mg^2+^ concentration arrests folding. Leave the arrested folding reactions at RT until adding substrate to initiate cleavage.
(4) Add native ribozyme (3.6 μL) from step 1 to a different cleavage buffer solution so that, after mixing, the mixture contains the exact same final concentrations and components as the mixture of folding time points and cleavage buffer from step 3. To achieve these conditions, the components present in the folding buffer (i.e., ATP-Mg^2+^ and chaperone protein or protein buffer) must be supplied directly in this cleavage buffer.
(5) Add 6FAM-rSA_5_-BHQ1 substrate (2 μL) to the unique mixtures present after steps 3 and 4. This initiates cleavage (cleavage time, *t*_2_ = 0). Collect one 6 μL time point from each of these reactions at a time dictated by the concentration of ribozyme present, as detailed in the Methods. Add the time point to 16 μL of 100 mM EDTA pH 8 to sequester Mg^2+^ and stop the substrate cleavage reaction. Leave the arrested cleavage reactions at RT until 20 μL is loaded to a 384-well microplate and run on a plate reader. If loading will occur several hours or the day after arresting cleavage, store the arrested cleavage reactions in the dark at 4 °C.

## Supporting Information Available

Validation experiment, detailed reaction schematics/components, and SpectraMax data (PDF)

## Abbreviations

6FAM: 6-carboxyfluorescein
BHQ1: black hole quencher 1
TE: tris ethylenediaminetetraacetic acid
NBS: nonbinding surface
