# Supplementary material for: Tracking Native Tetrahymena Ribozyme Folding with Fluorescence
Source: Biochemistry. 2023 Nov 1;62(22):3173–80. doi: 10.1021/acs.biochem.3c00363 (PMC10666665; doi:10.1021/acs.biochem.3c00363)
Supplement: Supplementary file 1 — bi3c00363_si_001.pdf [file bi3c00363_si_001.pdf]

Tracking Native *Tetrahymena* Ribozyme Folding with Fluorescence

Jeffrey P. Potratz and Rick Russell*

Department of Molecular Biosciences, University of Texas at Austin, Austin, Texas 78712, United States

***Corresponding Author**

Present Address: Department of Physical Sciences, Concordia University Wisconsin 12800 North Lake Shore Drive, Mequon, Wisconsin 53097, United States

E-mail: jeffrey.potratz@cuw.edu

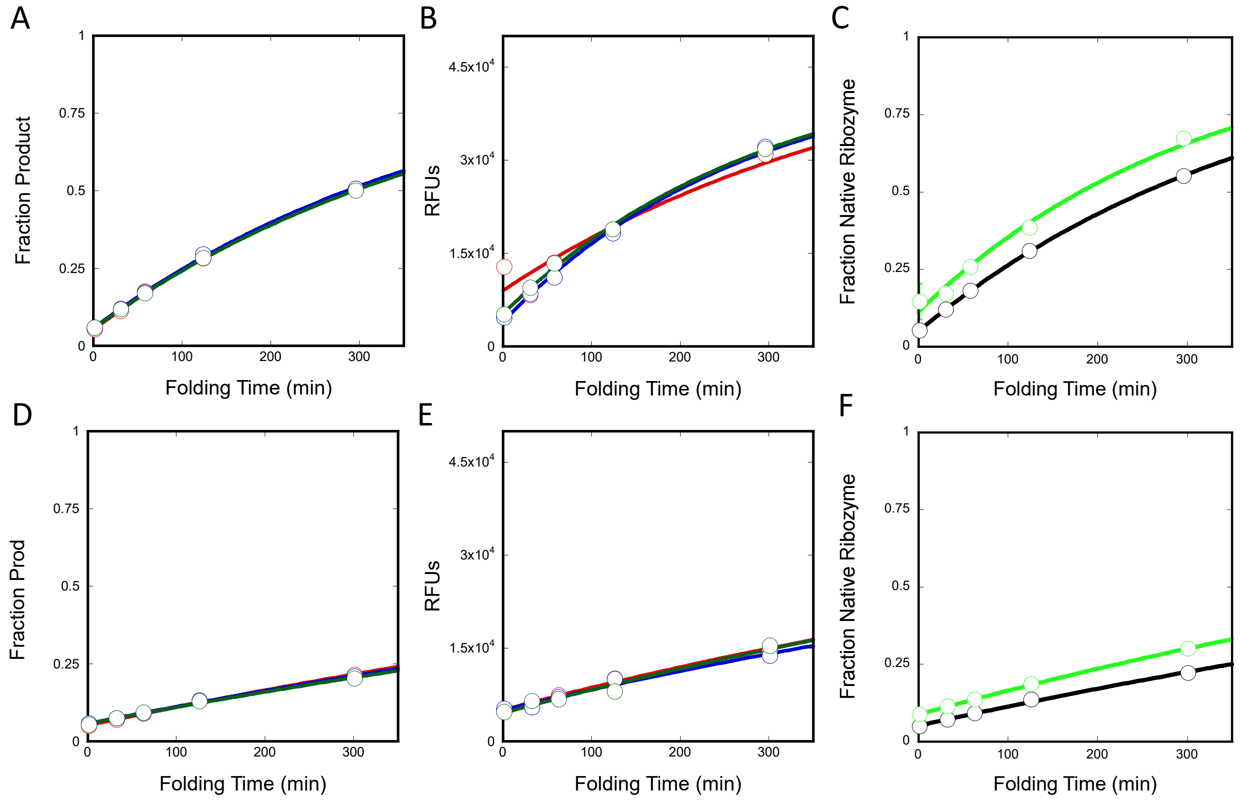

Figure S1. Validation experiment. A,B,C) Folding reactions with 5 mM Mg^{2+} and D,E,F) with 7 mM Mg^{2+}. A,D) Triplicate reactions (blue, red, green) with 60 nM ribozyme and ≤ 1 nM radiolabeled substrate and B,E) triplicate reactions (blue, red, green) with 60 nM ribozyme and 50 nM fluorescent substrate. Raw data from the radioactivity-based assay (A,D) and the fluorescence assay (B,E) were converted into fraction native ribozyme (C,F). An average value from triplicate SC+ (substrate control plus Ca^{2+}-folded ribozyme) reactions signifying 0% native ribozyme was subtracted from raw data. Then the data were normalized by an average value from triplicate FC (folded control) reactions signifying 100% native ribozyme. In C and F triplicate radioactivity-based assay data (black) time points were displayed as the average and standard error and the fluorescence data (bright green) were displayed similarly for comparison. Note the folding time courses are not complete within the observation time and the end points of the exponential fits were dictated by values obtained in control reactions for A,B,D,E and set to 1 for C and F.

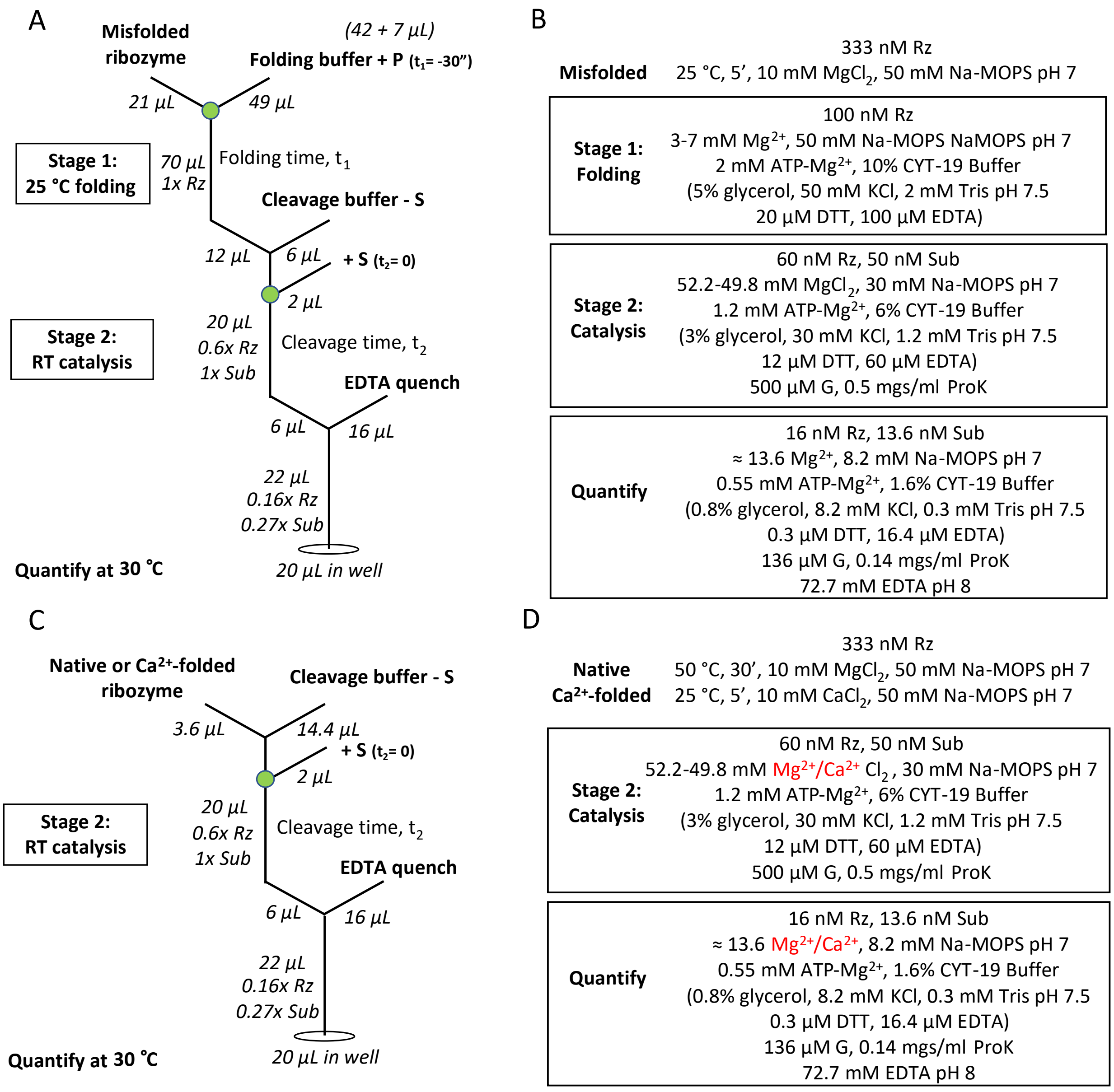

Figure S2. Detailed assay overview. A) Work flow for folding reactions and C) control reactions FC and SC+. B) Solution composition for the different stages of the assay for folding reactions and D) control reactions FC and SC+. Either Mg^{2+} or Ca^{2+} were included. Green dots indicate when stages 1 and 2 begin. Concentrations in the stages reflect concentrations used in the validation experiment where the fluorescent assay was compared to the radioactive assay. P = protein; S = substrate; RT = room temperature

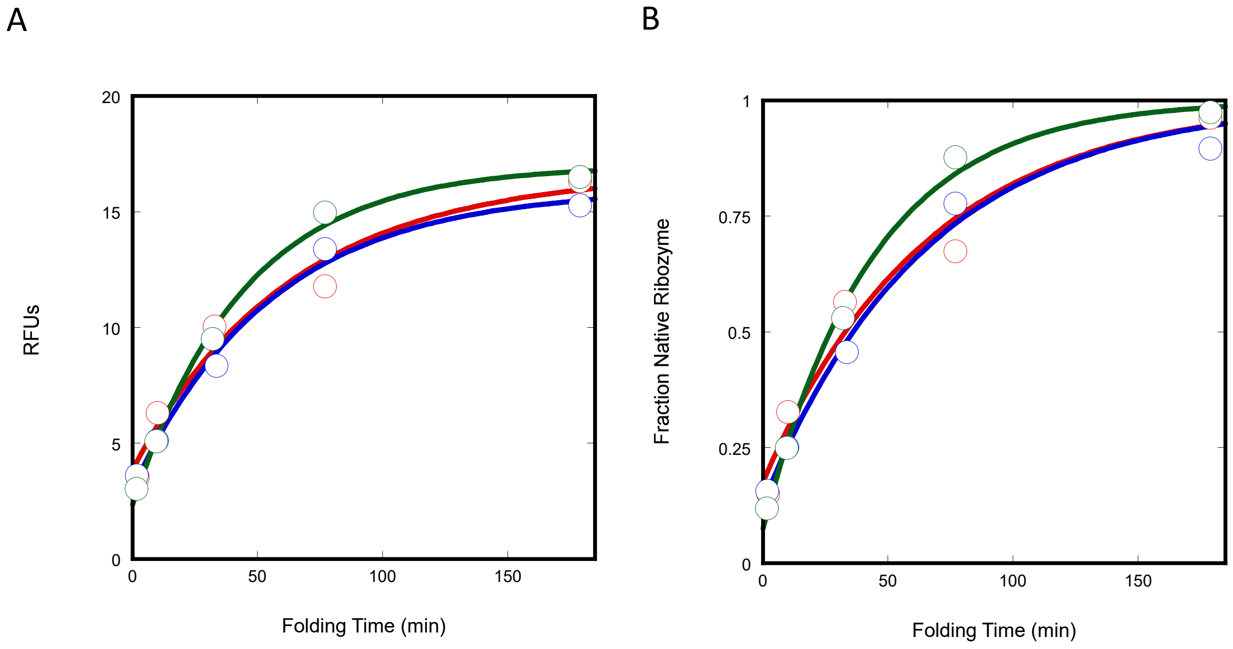

Figure S3. Data from SpectraMax M5. Folding reactions with 3 mM Mg^{2+}, 60 nM ribozyme, and 50 nM substrate. A) Raw data obtained with the SpectraMax M5 plate reader. B) Processed data using SC+ and FC control reactions.
